# Assessing current temporal and space-time anomalies of disease incidence

**DOI:** 10.1371/journal.pone.0188065

**Published:** 2017-11-13

**Authors:** Chih-Chieh Wu, Chien-Hsiun Chen, Sanjay Shete

**Affiliations:** 1 Department of Environmental and Occupational Health, College of Medicine, National Cheng Kung University, Tainan, Taiwan; 2 Institute of Biomedical Sciences, Academia Sinica, Taipei, Taiwan; 3 School of Chinese Medicine, China Medical University, Taichung, Taiwan; 4 Department of Biostatistics, The University of Texas MD Anderson Cancer Center, Houston, Texas, United States of America; Uniformed Services University of the Health Sciences, UNITED STATES

## Abstract

Approaches used to early and accurately characterize epidemiologic patterns of disease incidence in a temporal and spatial series are becoming increasingly important. Cluster tests are generally designed for retrospective detection of epidemiologic anomalies in a temporal or space-time series. Timely identification of anomalies of disease or poisoning incidence during ongoing surveillance or an outbreak requires the use of sensitive statistical methods that recognize an incidence pattern at the time of occurrence. This report describes 2 novel analytical methods that focus on detecting anomalies of incidence at the time of occurrence in a temporal and space-time series. The first method describes the paucity of incidence at the time of occurrence in an ongoing surveillance and is designed to evaluate whether a decline in incidence occurs on the single current day or during the most recent few days. The second method provides an overall assessment of current clustering or paucity of incidence in a space-time series, allowing for several space regions. We illustrate the application of these methods using a subsample of a temporal series of data on the largest dengue outbreak in Taiwan in 2015 since World War II and demonstrate that they are useful to efficiently monitor incoming data for current clustering and paucity of incidence in a temporal and space-time series. In light of the recent global emergence and resurgence of Zika, dengue, and chikungunya infection, these approaching for detecting current anomalies of incidence in the ongoing surveillance of disease are particularly desired and needed.

## Introduction

Approaches used to early and accurately characterize epidemiologic patterns of disease incidence in a temporal and spatial series are becoming increasingly important. Statistical analysis for detecting temporal and space-time anomalies (clusters and paucity) of health-related events is often required for various epidemiologic and biomedical applications. Cluster tests are generally designed for retrospective detection of epidemiologic anomalies in a temporal or space-time series. Timely identification of anomalies of disease or poisoning incidence during ongoing surveillance or an outbreak requires the use of sensitive statistical methods that recognize an incidence pattern at the time of occurrence. However, clusters or outbreaks are usually detected when the specialists or professionals of health agencies notice an unusually high frequency of health-related events. Perceptions of clustering or decline in incidence are often intuitive without statistical analysis. However, what is necessary is to determine whether or not a cluster or decline in incidence occurs to an extent greater than what would be expected by chance variation.

Hryhorczuk *et al*. discussed the importance of enhancing early detection and suggested using the scan test to detect temporal clustering of poisoning cases in the data reported daily to poison control centers [1]. The scan test employs a moving window of predetermined length *w* and finds the maximum number of cases revealed through the window as it slides over the entire region [2]. The scan test is structured to detect the largest cluster of incidence. The maximum number of events occurring in a window is the test statistic for the scan test. The authors demonstrated that the scan test can be retrospectively applied in the daily surveillance of poisoning clusters in an analysis of the temporal clustering of carbon monoxide poisonings. In contrast to detecting historical clusters, Grimson *et al*. proposed a statistical method that is sensitive to detect clusters in incidence at the time of occurrence [3]. Their method, based on a binomial distribution, is designed to detect current clusters of incidence with a duration of one or more days during an ongoing daily data collection and monitoring process. The authors applied their method to the daily carbon monoxide poisoning incidence data on which the scan test had been applied in the study by Hryhorczuk *et al*., and showed that their test for current clusters of incidence evidently has more power than the scan test.

The purpose of this report is to illustrate the use of novel statistical approaches that focus on detecting anomalies of incidence at the time of occurrence in a temporal series and in a space-time series. In contrast with the existing method for current clustering [3], one approach is designed to detect paucity of health-related events on the single current day of occurrence or on the most recent few days in a temporal series. When several space regions are involved, we suggest a global form of the test for detecting current clusters or paucity of incidence in a space-time series, depending on the alternative hypothesis. The global test is similar in construction to the Ederer-Myers-Mantel (EMM) test for space-time clustering based on the maximum frequency in a unit of time [4], the ***V*** test for space-time vacuity based on the minimum frequency in a unit of time [5], and the scan test for space-time clustering [6]. The global test for incidence anomalies provides an overall assessment, allowing for several space regions in the setting, and has more power for detecting anomalies than do the tests to investigate observed overall temporal incidence combined across multiple space regions.

Testing for excessive aggregations of disease incidence that occurred during a single current unit of time (e.g., day, week) or most recent few consecutive units of time is used to signal the occurrence of an excess of incidence in the current time period, permitting the immediate response and application of early intervention. In contrast, the detection of unusually sparse incidence of disease at the time of occurrence characterizes the current disease activity and epidemiologic transmission in an opposite way. A small p-value of the test indicates that a decline in disease incidence is occurring within the current time period, allowing for immediate assessment of an intervention strategy and decisions regarding prevention programs in the ongoing daily monitoring process.

We illustrate these methods using a subsample of a temporal series of data on dengue incidence in 2015 from the Taiwan Centers for Disease Control and demonstrate that they are useful to efficiently monitor incoming data for clustering and paucity of incidence in a temporal and space-time series. With the recent global emergence and resurgence of epidemic arboviruses such as Zika, dengue, and chikungunya, statistical methods for detecting current anomalies in the ongoing surveillance of disease are particularly desired and needed.

Most of the analytic methods for spatial and temporal analysis proposed in the statistical and epidemiological literature are retrospective in nature, particularly those for spatial analysis. Several prospective analytic methods for early detection of emerging disease outbreaks were developed recently, including those used in a temporal series [7–11] and those used in a space-time series [12]. These prospective analytic methods in a temporal series are designed to identify disease outbreaks over a broad geographical area (e.g., country) and are useful when relatively few cases are observed in any one jurisdiction. They usually require knowledge or assumptions of probability distributions that underlie the data and may need exploratory studies or preliminary analysis for the estimation of model parameters. In contrast, the analytic methods that we proposed here require mild assumptions with the null hypothesis of randomization and are structured to assess whether the incidence during the current few days progresses at the same rate, at a higher rate, or at a lower rate within a surveillance period. The spatial scan test for time periodic geographical disease surveillance by Kulldorff (2001) are designed to detect geographical disease clusters that remain during the last time period for which data are available [12]. While the global form of the proposed test for detecting current clusters or paucity of incidence when several space regions are involved is purely temporal in nature without involvement with the detection of geographical disease clusters.

## Methods

Suppose that *K* health-related events have occurred during *T* days. Consider the frequency of health-related events that occurred within the most recent *w* days in comparison with the frequency of health-related events that occurred in the *T—w* previous days. What interests us is to determine whether the observation of *x* events that occurred on the current day or during the most recent *w* days is rare compared with the occurrence of *K*—*x* events during the *T*—*w* previous days. Assuming that *X* is the random variable that represents the number of events occurring within the most recent *w* days and that each of the *K* events independently and equally occurs on one of the *T* days, the test for current paucity of incidence, denoted by ***Pau***, is based on the random variable *X* with a binomial distribution. The exact p-value formula for ***Pau*** under the null hypothesis of random allocations of *K* health-related events over the *T* days is expressed as follows:

$$P(X\leq x|K,T,w;p_{0}=\frac{w}{T})=\sum_{i\leq x}\left(\begin{array}{c} K\\ i \end{array}\right)p_{0}^{i}{\left(1-p_{0}\right)}^{K-i},$$ (1)

where *x* represents the observed number of events within the most recent *w* days. The test ***Pau*** assesses the significance of the small frequency at the time of occurrence on the basis of randomization and is used to measure an empirical paucity of incidence within the most recent *w* days. A small p-value of Expression (1) indicates that the observed *x* events occurring within the most recent *w* days, compared with the frequency of health-related events occurring during the *T*—*w* previous days, is significantly sparse; that is, a decline in incidence in the current *w*-day period occurs within the *T*-day surveillance period. Expression (1) gives an exact p-value of the test ***Pau*** for current paucity of incidence on the single current day for *w* = 1 and on the most recent 3 days for *w* = 3.

The exact p-value formula of the test for current clusters by Grimson et al [3], denoted by ***Clu***, is as follows:

$$P(X\geq x|K,T,w;p_{0}=\frac{w}{T})=\sum_{i\geq x}\left(\begin{array}{c} K\\ i \end{array}\right)p_{0}^{i}{\left(1-p_{0}\right)}^{K-i},$$ (2)

where *x* represents the observed number of events within the most recent *w* days. Expression (2) measures an empirical cluster of incidence at the time of occurrence. A small p-value of Expression (2) indicates that the occurrence of *x* events that excessively aggregated within the most recent *w* days cannot be explained by chance alone. Expression (2) gives an exact p-value of the test ***Clu*** for current clustering of incidence on the single current day for *w* = 1 and on the most recent 3 days for *w* = 3. The tests ***Pau*** and ***Clu*** are based on the same binomial random variable but characterize opposite aspects of an observed incidence pattern at the time of occurrence. They are structured to respectively detect current paucity and current clustering of incidence in a temporal series.

When many space regions are involved, we suggest using the global form of the tests to detect current clustering or paucity of incidence in a space-time series. Letting *X*_*i*_ be the random variable that represents the number of events that occurred within the most recent *w* days in the *i*-th space unit and letting **E**(*X*_*i*_) and **Var**(*X*_*i*_) denote the expected value and variance of *X*_*i*_, respectively, the test ***M*** is defined as

$$M=\frac{\sum_{i=1}^{R}\left\{X_{i}-E\left(X_{i}\right)\right\}}{\sqrt{\sum_{i=1}^{R}Var\left(X_{i}\right)}},$$ (3)

If the total number of space units, *R*, is large and the involved space units are relatively homogeneous in size, then the global test, ***M***, approximately follows a standard normal distribution under the null hypothesis of random arrangements of health-related events in each of the space units. The test ***M*** provides an overall assessment of current clustering or paucity of incidence in a space-time series, depending on the alternative hypothesis of ***M*** > 0 or ***M*** < 0. A large positive value of the test statistic ***M*** indicates that an excess of disease incidence occurs within the current time period in the time line for several geographically described population (e.g., hospitals, towns, or counties). In contrast, a small negative value of ***M*** indicates that a decline in incidence occurs within the current time period for several geographically described population.

Letting *Max*_*i*_ be the maximum frequency in a unit of time in the *i*-th space-time unit, the EMM test [13] is expressed as $EMM\;=\;\sum_{i\;=\;1}^{R}\left\{{Max}_{i}-E({Max}_{i})\right\}/\sqrt{\sum_{i\;=\;1}^{R}Var({Max}_{i})}.$ Replacing *Max* with *Min* in the above expression is the test ***V*** for space-time vacuity based on the minimum frequency that developed in a unit of time [5].

## Applications of the tests to dengue outbreak data

**Fig 1:**
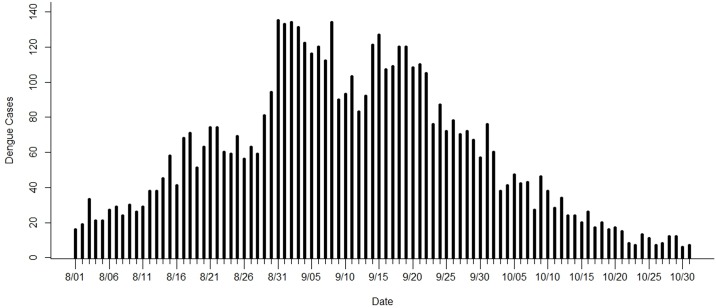
Daily dengue incidence data for North District, Tainan city, Taiwan, from August to October, 2015.

Dengue fever is the most common arbovirus infection in humans with virus transmission occurring in more than 100 countries in tropical regions. It is estimated that 390 million dengue infections occur annually, of which 50–100 million cases have apparent clinical manifestations [14–16]. The data on dengue incidence from Taiwan provide an opportunity to illustrate the applications of these methods for detecting current temporal and space-time anomalies of incidence. Dengue fever is a notifiable communicable disease in Taiwan. Information on dengue cases collected in Taiwan since 1988 is publicly available through the Taiwan Centers for Disease Control (http://www.cdc.gov.tw/english/index.aspx) and the Taiwan Government Open Data website (http://data.gov.tw/). This information includes the date an individual was diagnosed with dengue infection, his or her residence at diagnosis, place of infection, gender, and age. The largest dengue outbreak in Taiwan since World War II occurred in 2015. There were 43,419 confirmed autochthonous cases in Taiwan in 2015, among which 53% (22,842 cases) occurred in Tainan city, which is located in the southern, tropical region of Taiwan. For the purpose of illustration, we selected a subsample of dengue incidence data in Tainan and analyzed a temporal series of data on dengue incidence from August to October 2015. Fig 1 displays the daily dengue incidence data for Tainan’s North District from August to October 2015. The first dengue case in Tainan was reported in North District at the end of May 2015. A district is an administratively defined subdivision of a city in Taiwan and has its own health department that regularly reports health information to the city government. Tainan comprises 37 districts.

### Testing for current temporal clustering and paucity of incidence

We illustrate the use of the tests ***Clu*** and ***Pau*** for current clustering and paucity of dengue incidence, respectively, in the ongoing surveillance using the daily data in Fig 1 as follows. Where a day is the unit of time, setting *w* = 3 and *T* = 10, the number of cases reported during the most recent 3 days are compared with the number of cases that occurred in the previous 7 days. On the 10^th^ day of August, 80 cases that occurred during the most recent 3 days (that is, August 8–10) are compared with 166 cases that occurred during the 7 previous days (August 1–7). Using Expression (2), the exact p-value for current 3-day clusters of incidence is not significant on August 10 with *P(X ≥ 80| K = 246*, *T = 10*, *w = 3; p = 0*.*3) = 0*.*213*. The p-values for current 3-day clusters of incidence on August 11–15 are shown as follows:

August 11: *P(X ≥ 85| K = 259*, *T = 10*, *w = 3; p = 0*.*3) = 0*.*178*,
August 12: *P(X ≥ 93| K = 278*, *T = 10*, *w = 3; p = 0*.*3) = 0*.*117*,
August 13: *P(X ≥ 105| K = 283*, *T = 10*, *w = 3; p = 0*.*3) = 6*.*18×10*^*−3*^,
August 14: *P(X ≥ 121| K = 307*, *T = 10*, *w = 3; p = 0*.*3) = 2*.*73×10*^*−4*^,
August 15: *P(X ≥ 141| K = 344*, *T = 10*, *w = 3; p = 0*.*3) = 9*.*65×10*^*−6*^.

A small p-value of 6.18×10^−3^ on August 13 indicates that an excess of dengue cases for current 3-day clusters is identified within the 10-day surveillance period at a nominal significance level of 0.05; that is, an excess of dengue cases has occurred in the most recent 3-day period. Low p-values on August 13, 14, and 15 reflect the high incidence on those days in comparison with the incidence that occurred during the previous few days, indicating that the dengue incidence becomes worse daily, as shown in Fig 1.

Next, we illustrate the use of Expression (1) to evaluate the evidence of a decline in dengue incidence in the daily monitoring process. On the 22^nd^ of September, 323 cases that occurred during the most recent 3 days (September 20–22) are compared with 796 cases that occurred during the 7 previous days (September 13–19). The exact p-value for current 3-day paucity of incidence on September 22 is not significant with *P(X ≤ 323| K = 1119*, *T = 10*, *w = 3; p = 0*.*3) = 0*.*214*, based on Expression (1). The p-values for current 3-day paucity of incidence on September 23–26 are as follows:

September 23: *P(X ≤ 291| K = 1103*, *T = 10*, *w = 3; p = 0*.*3) = 4*.*45×10*^*−3*^,
September 24: *P(X ≤ 268| K = 1069*, *T = 10*, *w = 3; p = 0*.*3) = 2*.*01×10*^*−4*^,
September 25: *P(X ≤ 235| K = 1014*, *T = 10*, *w = 3; p = 0*.*3) = 7*.*18×10*^*−7*^,
September 26: *P(X ≤ 237| K = 986*, *T = 10*, *w = 3; p = 0*.*3) = 1*.*95×10*^*−5*^.

On September 23, a small p-value of 4.45×10^−3^ for current 3-day paucity of incidence is obtained, indicating that a decline in dengue incidence during the current 3-day period has occurred within the 10-day surveillance period. Low dengue incidence on September 23–26, compared with the incidence that occurred during the previous few days, results in very small p-values of Expression (1), indicating that the dengue incidence declines daily, as shown in Fig 1.

### Testing for current space-time clustering and paucity of incidence

**Fig 2:**
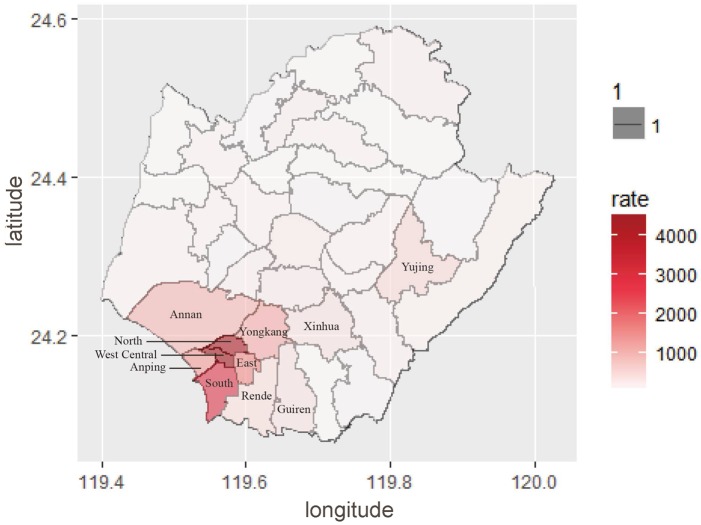
District-specific dengue incidence intensity map in 2015 Tainan.

We selected 11 districts in Tainan with the highest dengue rates to illustrate the testing for current clustering and paucity of incidence in a space-time series, using the test ***M*** shown in Expression (3). The rates, which were the numbers of dengue cases per 100,000 persons, ranged from 0 to 4,497 among the 37 districts in Tainan in 2015. The 11 districts with the highest rates were West Central (rate of 4,497), North (4,313), South (2,785), East (1,673), Anping (1,401), Yongkang (1,159), Annan (984), Yujing (480), Rende (422), Xinhua (358), and Guiren (315). The remaining 26 districts had a rate of 202 or less. Fig 2 presents the district-specific dengue incidence intensity in Tainan in 2015.

For the purpose of illustration, we analyzed a temporal series of dengue incidence data over these 11 districts on August 1–15 and September 13–26, 2015, which are the same time periods as those in the analysis above using ***Clu*** and ***Pau***. Where a day is the unit of time, *w* = 3, *T* = 10, and *R*, the total number of space units, is 11 in the model conditions. We obtained ***M*** = 1.29 on August 10, which gives a p-value of 0.098 and does not reject the null hypothesis of randomization at a 0.05 nominal significance level. The test statistics and p-values of ***M*** for current 3-day clusters in the 10-day surveillance period on August 11–15 are as follows:

August 11: ***M*** = 1.91 with a p-value of 2.79×10^−2^,
August 12: ***M*** = 1.51 with a p-value of 6.53×10^−2^,
August 13: ***M*** = 2.79 with a p-value of 2.66×10^−3^,
August 14: ***M*** = 3.56 with a p-value of 1.83×10^−4^,
August 15: ***M*** = 5.27 with a p-value of 6.85×10^−8^.

Small p-values (< 0.05) for current 3-day clusters are identified on August 11 and August 13–15, indicating that there exists a significantly important departure from the expected frequencies and that the dengue incidence becomes worse each of these days over these 11 districts.

Next, we illustrate the use of the ***M*** test to evaluate the evidence of a space-time decline in dengue incidence within the ongoing daily surveillance. In the same settings of *w* = 3, *T* = 10, and *R* = 11, ***M*** is applied to the data on September 22. We obtain ***M*** = 1.11, which gives a p-value of 0.886, and do not reject the null hypothesis of randomization at a 0.05 nominal significance level. No decline in dengue incidence is detected over these 11 districts on September 22. The p-values for current 3-day paucity of incidence on September 23–26 in the 10-day surveillance period are presented as follows:

September 23: ***M*** = -2.76 with a p-value of 2.87×10^−3^,
September 24: ***M*** = -6.66 with a p-value of 1.39×10^−11^,
September 25: ***M*** = -9.53 with a p-value of 7.28×10^−22^,
September 26: ***M*** = -10.06 with a p-value of 4.30×10^−24^.

The very small p-values presented above show that a space-time decline in dengue incidence is detected on September 23–26. We note that the size of the decline increases daily over the 4-day period. The global test ***M*** is more powerful for detecting temporal anomalies in incidence than do ***Pau*** and ***Clu*** to investigate observed overall annual incidence combined over the 11 districts because the global test uses temporal incidence information for each of the 11 districts [4–6].

## Discussion

Cluster tests are generally designed for retrospective detection of epidemiologic anomalies over a temporal or space-time series. Existing tests for clustering that focus on detecting the times of large or “peak” incidence in a temporal or space-time series, including the EMM test [4,13], scan test [2,6], and Maxima test [17,18], are all based on the maximum frequency in a unit of time. We and others have developed tests that focus on the times of paucity of events, including the empty cells (or empty columns) test [17,18], run of empty cells tests [19], and Minima and ***V*** tests that are based on the minimum frequency in a unit of time [5,20]. In this report, we illustrated the use of novel statistical approaches to testing of significance for the small or large frequencies at the time of occurrence on the basis of random allocations of distinct epidemiologic events into consecutive days, which underlies a binomial distribution. The first approach uses a test that describes the paucity of disease incidence at the time of occurrence in an ongoing surveillance and is designed to evaluate whether a decline in incidence occurs on the single current day of occurrence or during the most recent few days, which is the test ***Pau***. In contrast, the existing test, ***Clu***, represents a direct measure of clustering as many cases accumulating at the time of occurrence and is used to assess the evidence of whether an excess of disease incidence has occurred on the single current day or during the most recent few days [3]. The second approach uses the global form of the tests in a temporal series to test for current clustering or paucity of incidence in a space-time series, depending on the alternative hypothesis of ***M*** > 0 or ***M*** < 0, which is the ***M*** test.

***Pau*** and ***Clu***, which are structured to characterize current disease activity and epidemiologic transmission in opposite ways, are useful to determine whether a decline or cluster in incidence has occurred on the single current day or during the most recent few days in the ongoing daily monitoring process, respectively. Statistical approaches that are sensitive to current paucity or clustering of incidence in a temporal and space-time series as presented here provide early and accurate recognition and identification of clusters and declining incidence, which are required for application and assessment of early intervention strategies and for effective disease prevention and control [21]. For instance, health authorities may expand (or change) an intervention strategy as soon as a decline in incidence is (or is not) detected after the use of certain insecticide sprays in a given region. When an excess of disease incidence is identified at a time point, intervention can be initialized immediately. Climate factors, such as sudden cold spells or heat waves, may instantly affect disease activity and epidemiologic transmission. The use of the tests, ***Pau***, ***Clu***, and ***M***, allows health authorities or investigators to statistically evaluate the association between the local climate variables and disease incidence. Diseases for which activity and transmission are affected by environmental or climatic factors are particularly modifiable by intervention.

The use of daily testing in an ongoing surveillance process raises the issue of multiple comparisons. Procedures, such as Bonferroni and False Discovery Rate corrections, have been developed for controlling false positives by using a smaller nominal significance level (< 0.05) for rejecting the null hypothesis [22]. While the use of these adjustments also reduces the overall statistical power and may miss true positives. More importantly, Mantel and others emphasize that the purpose of applying the tests in a monitoring process is “signaling” rather than hypothesis testing [1,3,13,23–25]. Therefore, the use of multiple comparison procedures is not recommended here. In the applications to dengue incidence above, we set *T* = 10 (surveillance period) and *w* = 3 (current period). The choice of the appropriate size of unit scale in time for testing anomalies of incidence depends on the disease, the frequency and duration of an outbreak, and perhaps other considerations. We suggest using various sets of the values of w and T for further charactering and comparing the temporal patterns of incidence at the time of occurrence, particularly when the knowledge of the disease etiology is lacking. In the application for daily ongoing surveillance of infectious diseases such as dengue or Zika, one may consider a smaller number for w (e.g., 1 or 3) and T (e.g., 7 or 10). In interpretation of the outcomes of the analysis, we must emphasize that the results are based on the specific sizes of unit scales in time or space chosen.

Recently, Kulldorff (2001) stressed that p-values should be used as an indicator concerning the evidence for true clustering and the amount of effort for the investigation should be dependent on this evidence rather than maintaining a strict cut-off for the p-value to determine detected clusters to be investigated or not [12]. In addition to using multiple comparison adjustments, understanding the correlation between the proposed tests on the two consecutive days will be very helpful for controlling false signal rates. This correlation structure is complex and warrants future research.

The recent global emergence of Zika virus infection and its severe forms, Guillain-Barre syndrome and microcephaly, which have been associated with the Zika virus in French Polynesia and Brazil, suggest that Zika has become a very serious global public health problem [26]. Active disease surveillance is designed to monitor disease activity and epidemiologic transmission. Health authorities must be able to accurately determine whether a decline or cluster in incidence is happening at the time of disease occurrence [21,27]. Statistical methods to efficiently monitor incoming data for clustering and paucity of incidence in a temporal and space-time series as presented here are increasingly desired in light of the recent global emergence of Zika and dengue infection.

## References

[1] Hryhorczuk DO, Frateschi LJ, Lipscomb JW, Zhang R (1992) Use of the scan statistic to detect temporal clustering of poisonings. Journal of Toxicology: Clinical Toxicology 30: 459–465. 1512818 10.3109/15563659209021560

[2] Wallenstein S, Neff N (1987) An approximation for the distribution of the scan statistic. Statistics in Medicine 6: 197–207. 3589249 10.1002/sim.4780060212

[3] Grimson RC, Mendelsohn S (2000) A method for detecting current temporal clusters of toxic events through data monitoring by poison control centers. Journal of Toxicology: Clinical Toxicology 38: 761–765. 11192463 10.1081/clt-100102389

[4] Ederer F, Myers MH, Mantel N (1964) A statistical problem in space and time: do leukemia cases come in clusters? Biometrics 20: 626–638.

[5] Wu CC, Grimson RC, Amos CI, Shete S (2008) Statistical methods for anomalous discrete time series based on minimum cell count. Biometrical Journal 50: 86–96. doi: 10.1002/bimj.200610374 17853406 10.1002/bimj.200610374

[6] Wallenstein S, Gould MS, Kleinman M (1989) Use of the scan statistic to detect time-space clustering. American Journal of Epidemiology 130: 1057–1064. 2816892 10.1093/oxfordjournals.aje.a115406

[7] Farrington CP, Andrews NJ, Beale A, Catchpole MA (1996) A statistical algorithm for the early detection of outbreaks of infectious disease. Journal of the Royal Statistical Society Series A (Statistics in Society): 547–563.

[8] Hutwagner L, Maloney EK, Bean NH, Slutsker L, Martin S (1997) Using laboratory-based surveillance data for prevention: an algorithm for detecting Salmonella outbreaks. Emerging infectious diseases 3: 395 doi: 10.3201/eid0303.970322 9284390 10.3201/eid0303.970322 PMC2627626

[9] Nobre FF, Stroup DF (1994) A monitoring system to detect changes in public health surveillance data. International journal of epidemiology 23: 408–418. 8082970 10.1093/ije/23.2.408

[10] Reis BY, Mandl KD (2003) Time series modeling for syndromic surveillance. BMC Medical Informatics and Decision Making 3: 2 doi: 10.1186/1472-6947-3-2 12542838 10.1186/1472-6947-3-2 PMC149370

[11] Sonesson C, Bock D (2003) A review and discussion of prospective statistical surveillance in public health. Journal of the Royal Statistical Society: Series A (Statistics in Society) 166: 5–21.

[12] Kulldorff M (2001) Prospective time periodic geographical disease surveillance using a scan statistic. Journal of the Royal Statistical Society: Series A (Statistics in Society) 164: 61–72.

[13] Mantel N, Kryscio RJ, Myers MH (1976) Tables and formulas for extended use of the Ederer-Myers-Mantel disease-clustering procedure. American Journal of Epidemiology 104: 576–584. 984032 10.1093/oxfordjournals.aje.a112333

[14] Bhatt S, Gething PW, Brady OJ, Messina JP, Farlow AW, Moyes CL, et al (2013) The global distribution and burden of dengue. Nature 496: 504–507. doi: 10.1038/nature12060 23563266 10.1038/nature12060 PMC3651993

[15] Stanaway JD, Shepard DS, Undurraga EA, Halasa YA, Coffeng LE, Brady OJ, et al (2016) The global burden of dengue: an analysis from the Global Burden of Disease Study 2013. The Lancet Infectious Diseases. 10.1016/S1473-3099(16)00026-8 PMC5012511 26874619

[16] Wilder-Smith A, Gubler DJ (2015) Dengue vaccines at a crossroad. Science 350: 626–627. doi: 10.1126/science.aab4047 26542552 10.1126/science.aab4047

[17] Grimson RC (1993) Disease clusters, exact distributions of maxima, and P-values. Statistics in Medicine 12: 1773–1794. 8272660 10.1002/sim.4780121906

[18] Grimson RC, Oden N (1996) Disease clusters in structured environments. Statistics in Medicine 15: 851–871. 9132911 10.1002/(sici)1097-0258(19960415)15:7/9<851::aid-sim255>3.0.co;2-4

[19] Grimson RC, Aldrich TE, Drane JW (1992) Clustering in sparse data and an analysis of rhabdomyosarcoma incidence. Statistics in Medicine 11: 761–768. 1594815 10.1002/sim.4780110607

[20] Wu CC, Grimson RC, Shete S (2010) Exact statistical tests for heterogeneity of frequencies based on extreme values. Communications in Statistics—Simulation and Computation 39: 612–623. doi: 10.1080/03610910903528335 25558124 10.1080/03610910903528335 PMC4282130

[21] Guzman MG, Halstead SB, Artsob H, Buchy P, Farrar J, Gubler DJ, et al (2010) Dengue: a continuing global threat. Nature Reviews Microbiology 8: S7–S16. doi: 10.1038/nrmicro2460 21079655 10.1038/nrmicro2460 PMC4333201

[22] Benjamani Y, Hochberg Y (1995) Controlling the False Discovery Rate: A Practical and Powerful Approach to Multiple Testing. Journal of the Royal Statistical Society Series B 57: 289–300.

[23] Jones DR, Rushton L (1982) Simultaneous inference in epidemiological studies. International Journal of Epidemiology 11: 276–282. 7129742 10.1093/ije/11.3.276

[24] Savitz DA, Olshan AF (1995) Multiple comparisons and related issues in the interpretation of epidemiologic data. Am J Epidemiol 142: 904–908. 7572970 10.1093/oxfordjournals.aje.a117737

[25] Thomas DC, Siemiatycki J, Dewar R, Robins J, Goldberg M, Armstrong BG (1985) The problem of multiple inference in studies designed to generate hypotheses. Am J Epidemiol 122: 1080–1095. 4061442 10.1093/oxfordjournals.aje.a114189

[26] Musso D, Gubler DJ (2016) Zika virus. Clinical Microbiology Reviews 29: 487–524. doi: 10.1128/CMR.00072-15 27029595 10.1128/CMR.00072-15 PMC4861986

[27] Gubler DJ (1998) Dengue and dengue hemorrhagic fever. Clinical Microbiology Reviews 11: 480–496. 9665979 10.1128/cmr.11.3.480 PMC88892

